# Crosstalking with dendritic cells: a path to engineer advanced T Cell immunotherapy

**DOI:** 10.3389/fsysb.2024.1372995

**Published:** 2024-04-29

**Authors:** Sogand Schafer, Kaige Chen, Leyuan Ma

**Affiliations:** ^1^ Center for Craniofacial Innovation, Children’s Hospital of Philadelphia Research Institute, Children’s Hospital of Philadelphia, Philadelphia, PA, United States; ^2^ Division of Plastic and Reconstructive Surgery, Department of Surgery, Children’s Hospital of Philadelphia, Philadelphia, PA, United States; ^3^ Department of Pathology and Laboratory Medicine, Perelman School of Medicine, University of Pennsylvania, Philadelphia, PA, United States; ^4^ Center for Cellular Immunotherapies, Perelman School of Medicine, University of Pennsylvania, Philadelphia, PA, United States; ^5^ Institute for Immunology, Perelman School of Medicine, University of Pennsylvania, Philadelphia, PA, United States; ^6^ Abramson Cancer Center, Perelman School of Medicine, University of Pennsylvania, Philadelphia, PA, United States; ^7^ The Raymond G. Perelman Center for Cellular and Molecular Therapeutics, Children’s Hospital of Philadelphia, Philadelphia, PA, United States

**Keywords:** dendritic cell, cellular crosstalk, immunological synapse, nanoparticles, biomaterials

## Abstract

Crosstalk between dendritic cells and T cells plays a crucial role in modulating immune responses in natural and pathological conditions. DC-T cell crosstalk is achieved through contact-dependent (i.e., immunological synapse) and contact-independent mechanisms (i.e., cytokines). Activated DCs upregulate co-stimulatory signals and secrete proinflammatory cytokines to orchestrate T cell activation and differentiation. Conversely, activated T helper cells “license” DCs towards maturation, while regulatory T cells (Tregs) silence DCs to elicit tolerogenic immunity. Strategies to efficiently modulate the DC-T cell crosstalk can be harnessed to promote immune activation for cancer immunotherapy or immune tolerance for the treatment of autoimmune diseases. Here, we review the natural crosstalk mechanisms between DC and T cells. We highlight bioengineering approaches to modulate DC-T cell crosstalk, including conventional vaccines, synthetic vaccines, and DC-mimics, and key seminal studies leveraging these approaches to steer immune response for the treatment of cancer and autoimmune diseases.

## 1 Introduction

As a specialized subset of antigen-presenting cells (APCs), DCs play a critical role in bridging the innate and adaptive immune response to initiate protective immunity against pathogens as well as to maintain tissue immune homeostasis (Rescigno and Sabatino, 2009; Møller et al., 2022). DCs exhibit remarkable functional diversity, underscored by their classification into two primary types: conventional DC1 (cDC1) and conventional DC2 (cDC2), possessing plasticity and the ability to respond to cues from various tissue microenvironments (Sigmundsdottir and Butcher, 2008; Cabeza-Cabrerizo et al., 2021). The crosstalk between DCs and effector immune cells, especially T cells, is a critical determinant of the immune response towards activation or tolerance (Fucikova et al., 2019).

DC-T cell crosstalk, crucial for immune responses, occurs through mechanisms that include immunological synapses and cytokine signaling (Hivroz et al., 2012). The immunological synapse is a specialized contact zone formed between DCs and T cells (Dustin, 2014). The initial contact zones on the surface of DCs are known as central supramolecular activation clusters (cSMAC), characterized by a coordinated array of antigen-presenting and co-stimulatory molecules (Dustin, 2014). Encircling the cSMAC are adhesion molecules like LFA-1, while the distal SMAC is composed of proteins with extensive extracellular domains, such as the phosphatases CD43 and CD45, which transmit suppressive signals (Alarcón et al., 2011). Within the cSMAC, major histocompatibility complexes (MHCs) present antigenic peptides to the T cell receptor (TCR) (Dustin, 2014), and the co-stimulatory molecules on DCs engage their cognate receptors on T cells (Frauwirth and Thompson, 2002). Notably, the quality of the immunological synapse is a critical determinant of T cell differentiation. A synapse formed from activated DCs are rich for co-stimulatory molecules, and their optimal clustering and strong TCR signaling collectively promote robust T cell activation and effector function (Snook et al., 2018; Solouki et al., 2019). Conversely, the absence or insufficient expression of co-stimulatory molecules on immature DCs, reduced secretion of proinflammatory cytokines, and weaker TCR signaling during antigen presentation can result in T cell anergy or the promotion of antigen-specific induced T regulatory cells (iTregs) (Schmitt and Williams, 2013; Li and Rudensky, 2016). These iTregs possess suppressive properties to further silence DCs, preventing naïve T cell activation in the immune response and promoting immune tolerance (Lafaille and Lafaille, 2009). The balance between activation and tolerance-inducing signals is critical for maintaining immune homeostasis. Finally, DCs secreted a myriad of cytokines such as interleukins, interferons, and tumor necrosis factors as key mediators in the orchestration of immune responses (Blanco et al., 2008). These soluble factors influence the differentiation, proliferation, effector functions, and polarization of T cells in a contact-independent manner (Dong, 2021).

In this review, we first discuss the natural crosstalk and bidirectional signaling events between DCs and T cells. Further, we cover the innovative bioengineering approaches to modulate the cellular crosstalk between DCs and T cells. In particular, we focus on vaccine engineering, a classical approach to tailor DC-T cell crosstalk, genetically modified DCs, and DC-mimicking nanostructures for controlled intervention of T cell differentiation, aiming at innovations in cancer and autoimmune therapeutics.

## 2 Natural DC-T cell crosstalk mechanisms

**FIGURE 1:**
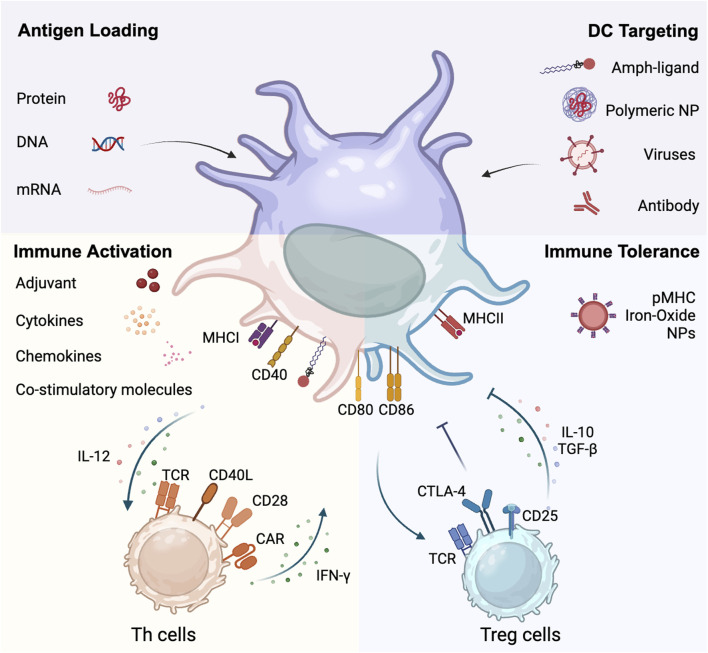
Dual role of dendritic cells in crosstalking with T cells. DCs can be modified and targeted by entities such as amph-ligands, polymeric nanoparticles, viruses, and antibodies. DCs processes and presents antigens derived from proteins, recombinant DNA and mRNA. DCs interface with both immune activation and tolerance pathways. On the immune activation side, DCs promote the differentiation of Th cells through upregulating surface proteins like MHCII, CD80, and CD86, and proinflammatory cytokines like IL-12. Conversely, activated Th cells upregulate CD40L and promotes DC maturation through the CD40L-CD40 axis. On the immune tolerance side, pMHC nanoparticles promotes Treg development. DCs engage with Treg cells, fostering an immunosuppressive milieu marked by Treg-associated cytokine production (IL-10 and TGF-β). This juxtaposition underscores the central role of DCs in orchestrating the balance between immune activation and tolerance.

The natural crosstalk between DC and naïve T cells leads to antigen-specific T cells priming and differentiation (Hivroz et al., 2012) (Figure 1). Co-stimulatory molecules, such as CD80 and CD86, on DCs engage with their cognate receptors, such as CD28 on T cells, providing additional signals for optimal T cell activation and effector differentiation (Frauwirth and Thompson, 2002). DCs can also crosstalk with antigen-experienced T cells or engineered T cells such as CAR T cells during antigen recall or vaccination (Zammit et al., 2005; Ma et al., 2019; Reinhard et al., 2020). Three signals from DCs are essential for optimal activation of naïve T cells: the first signal being the antigen, the second signal providing co-stimulation, and the third signal offering cytokine support (Curtsinger and Mescher, 2010).

### 2.1 Antigen presentation and recognition

DCs are specialized in sensing and processing antigens, initiating a cascade of molecular events through the recognition of pathogen-associated molecular patterns (PAMPs) or danger-associated molecular patterns (DAMPs) via pattern recognition receptors (PRRs) (Mogensen, 2009). DCs utilize mechanisms such as phagocytosis, macropinocytosis, or receptor-mediated endocytosis to capture PAMPs upon antigen encounter (Platt et al., 2010). Upon activation by pathogens, DCs initiate a maturation process. In this process, DCs upregulate their expression of costimulatory molecules and cytokines, reduce their phagocytic capability, and increase the transportation of MHC class II molecules from lysosomes to the DC surface (Platt et al., 2010; Dalod et al., 2014), which collectively make DCs more proficient in stimulating T cells (Platt et al., 2010).

Furthermore, captured antigens are processed into small peptides within endosomes and lysosomes (Blum et al., 2013; Embgenbroich and Burgdorf, 2018). These peptides are loaded onto MHC class I and II molecules on the DC surface and presented as trimeric peptide MHC complexes (pMHCs) to CD8^+^ T cells and CD4^+^ T helper (Th) cells, respectively (Blum et al., 2013). Notably, most of antigens presented on MHCs have an affinity of 1–100 µM toward the cognate TCR (Zhong et al., 2013). Therefore, efficient triggering of T cell activation is heavily dependent on co-receptors such as CD8 that stabilized interaction via an avidity effect (Laugel et al., 2007; Campillo-Davo et al., 2020), or CD4 that promotes signaling accumulation (Roh et al., 2015). It was found that weaker TCR signaling is prone to trigger memory development while stronger signaling can shift T cells differentiation toward effectors (Snook et al., 2018). However, during recall responses, memory T cells recognize antigens presented on DCs in a much more sensitive manner, with lower activation threshold than naïve T cells (Liu et al., 2020). This increased sensitivity or reduced activation threshold is likely due to the pre-formed TCR clusters on memory T cell surface (Kumar et al., 2011).

### 2.2 Co-stimulatory, co-inhibitory signals and cytokines

Among the key mechanisms of DC - T cell crosstalk are co-stimulatory and co-inhibitory signals (Cabeza-Cabrerizo et al., 2021). These co-regulatory signals, influenced by the DCs’ heterogeneity, lineage, maturation stage, and the tissue environment as well as the nature of the infectious agent play a pivotal role in tailoring immune responses (Hilligan and Ronchese, 2020). Co-stimulatory signals are transmitted through cell surface molecules, including members of both, the B7 family (e.g., CD80 and CD86) and the TNF receptor family (e.g., CD40) (Sharpe and Freeman, 2002). These molecules act as binding partners for receptors expressed on T cells such as CD28 and CTLA4 to modulate T cell activation and effector function (Sharpe and Freeman, 2002). CD28 is the most important co-stimulatory receptor due to its constitutive expression on naïve T cells, and engagement of CD28 with CD80/CD86 is the first co-stimulatory signal required for priming naïve T cells (Esensten et al., 2016). Upon T cell activation, CTLA4 expression will be induced and CD28 expression is concomitantly downregulated by endocytosis (Alegre et al., 2001). CTLA4 further competes for binding with CD80 and CD86 to relay inhibitory signals to T cells, limiting T cell activation by APCs (Alegre et al., 2001). A few other co-stimulatory and co-inhibitory receptors such as 41BB, ICOS, OX40, and PD-1 will also be rapidly induced within 24 h (Chen and Flies, 2013). These additional co-signaling receptors engage their cognate ligands on activated DCs, such as 41BBL, ICOSL, OX40L, and PD-L1/L2 respectively, promoting T cell expansion and differentiation or attenuating T cell activation (Chen and Flies, 2013).

In addition to co-regulatory receptors, cytokines play an indispensable role in supporting T cell activation as well as modulating T cell differentiation. IL-2 is the crucial cytokine supporting the activation of naïve T cells (Ross and Cantrell, 2018). DCs were found to produce IL-2 after bacteria uptake and this early time-point IL-2 production making DCs perfectly equipped to prime CD4 T cells (Granucci et al., 2001; Zelante et al., 2012). The remarkable plasticity of CD4 T cells allows them to respond to environmental stimuli and differentiate into a numerous Th cell subsets in a context-dependent manner (DuPage and Bluestone, 2016). For example, in the presence of a stronger TCR signaling, proinflammatory cytokines such as DC-derived IL-12 and natural killer cells-secreted IFN-γ will induce CD4 T cell differentiation to Th1 cells (Luckheeram et al., 2012). In the presence of suppressive cytokines, such as IL-4, CD4 T cell are prone to differentiate into Th2 cells (Luckheeram et al., 2012). IL-6 and IL-21 regulate Th17 cell development (Zhou et al., 2007; Luckheeram et al., 2012), and IL-10, TGF-β will induce Treg differentiation (Chen et al., 2003; Luckheeram et al., 2012).

Notably, this fundamental understanding of the DC-T cell crosstalk led to the development of chimeric antigen receptor (CAR) T cells that were engineered to recognize non-MHC-restricted antigens on diseased tissues such as cancer (Labanieh and Mackall, 2022; Neeser et al., 2023). This technique involves genetically modifying a patient’s T cells to express a CAR that specifically recognize a protein antigen on cancer cell surface. Once infused back into the patient, these engineered CAR T cells can effectively recognize and kill cancer cells. The innovation lies in its ability to bypass the conventional antigen presentation pathway, offering a potent and personalized therapeutic option against cancers previously deemed untreatable. Building on the various co-signaling domains and cytokines involved DC-T cell crosstalk, researchers have designed functionally enhanced next-generation CAR designs and armed CARs with significantly improved anti-tumor efficacy (Labanieh and Mackall, 2022). CAR T cell therapy has made remarkable stride in the treatment of relapsed B cell leukemia, lymphoma, and multiple myeloma (Mitra et al., 2023). Six different CAR T products have received FDA-approval since 2017 (Mitra et al., 2023), and many more preclinical and clinical CAR T trials are under way in various disease models, such as multiple solid tumors, type 1 diabetes, HIV infection, cardiac fibrosis, marking a new era of immunotherapy (extensively reviewed elsewhere (Baker et al., 2023; Neeser et al., 2023)).

### 2.3 T cell modulation of DCs

Activated T cells especially CD4 T cells upregulate CD40L which reversely signals to DCs through the CD40L-CD40 axis to activate DCs, a process known as “DC licensing” (Figure 1) (Elgueta et al., 2009; Ma and Clark, 2009). Licensed DCs enhance their expression of MHCII as well as co-stimulatory molecules including CD80/CD86, and increase the production of proinflammatory cytokines such as TNF-α, interferon-γ (IFN-γ), and interleukin-6 (IL-6) (Elgueta et al., 2009). This improvement in function makes them more effective as APCs, enabling them to more efficiently guide the differentiation of CD4 T cells towards Th1 cells and to prime CD8 T cells through the process of cross-presentation (Tay et al., 2017). Activated CD8 T cells acquire cytotoxic effector functions, with high expression level of cytotoxic molecules, such as granzymes, perforin and IFN-γ, poised to become professional killer cells for eradicating infected or malignant cells (Zhang and Bevan, 2011). Interestingly, a subset (30%–50%) of effector and memory CD8 T cells also upregulate CD40L (Frentsch et al., 2013; Tay et al., 2017). CD40L + CD8 T cells, in contrast to CD40L- CD8 T cells, share functional features with CD4 T helper cells as shown by their abilities to induce the maturation of monocyte-derived DCs. The presence of CD40L on CD8 T cells allows CD8 T cells to directly crosstalk with DCs such as in tumors without the need of CD4 T cells and to develop tissue-resident memory CD103+ CD8 T cells with enhanced anti-tumor immunity (Medler et al., 2023). Interestingly, CD19 CAR T cells equipped with CD40L “licensed” splenic DCs and macrophages as well as DCs in celiac and portal lymph nodes (LNs) in an A20 lymphoma model (Kuhn et al., 2019). The CD40L + CD19 CAR T cell therapy enhanced IL-12 production by splenic DCs and increased effector functions of endogenous non-CAR T cells (Kuhn et al., 2019). Despite the independent role of CD40L on CD4 or CD8 T cells during their crosstalk with DCs, a recent study from Schietinger’s group revealed a complex crosstalk mechanism among CD4^+^ T cells, CD8^+^ T cells, and DCs within the solid tumor microenvironment (Espinosa-Carrasco et al., 2023). The tricellular complex (i.e., triad) formed among CD4^+^ and CD8^+^ T cells and intratumoral DCs was demonstrated to promote functional reprogramming of adoptive transferred CD8 T cells, preventing or reversing exhaustion. The triad formation was found to be the most effective predictive marker of responses to immune checkpoint blockade (ICB) in mesothelioma (Espinosa-Carrasco et al., 2023).

Likewise, Tregs crosstalk with DCs in the periphery using both MHC-dependent or -independent mechanisms (Figure 1). Tregs have constitutive expression of CTLA4 which have higher affinity toward CD80/CD86 and tend to cluster around DCs (Maldonado and Andrian, 2010; Hasegawa and Matsumoto, 2018). Upon conjugation with DCs, Tregs sequester CD80/CD86 via CTLA4 molecules and deplete the complex from the DC surface, potentially by trogocytosis (Tekguc et al., 2021). Furthermore, Tregs constitutively express CD25 a high-affinity receptor subunit of IL-2 (Rudensky, 2011), making them highly efficient in consuming surrounding IL-2 molecules (Létourneau et al., 2009). In addition, Tregs-derived cytokines such as TGF-β and IL-10 suppress the maturation of DCs (Taylor et al., 2006). Collectively, these mechanisms make Tregs highly efficient in tolerizing DCs and preventing DCs from priming conventional T cells.

### 2.4 The spacing requirement of productive DC-T cell crosstalk

The synapse is differentially spaced depending on the functional zone. Following recognition of the cognate pMHC on DCs by the TCR, adhesion molecules such as LFA-1 and co-signaling receptors such as CD28 often co-localize with TCR molecules at the immunological synapse (Dustin, 2014). The distance between DC and T cell membranes is found to be generally 25–55 nm (Leithner et al., 2021), and the shorter TCR-pMHC pair translates to a closer contact ∼15 nM (Dustin and Depoil, 2011). This close contact within the synapse excluded inhibitory proteins such as phosphatase CD45 and CD148, which have a large extracellular domain of ∼50 nm (Cordoba et al., 2013). Without the need of *de novo* upregulation of positive signals or downregulation of negative signals, the biophysical segregation of signaling proteins into distinct domains enables local concentration of signaling molecules, favoring TCR activation in a highly precise and rapid manner. Dustin and colleagues dissected the impact of differential spacing on T cell activation by using CD45 or CD148 with truncated extracellular domains and observed diminished to complete abolishment of T cell activation, likely due to insufficient exclusion of the phosphatase activities of CD45 or CD148 (Cordoba et al., 2013). Inspired by the genetic approaches to modulate intermembrane spacing by altering the length of pMHC and other membrane proteins (James and Vale, 2012), Du et al. leveraged highly programmable DNA molecules to create membrane-anchored DNA nanojunctions. These DNA nanojunctions are capable of extending to desired sizes, maintaining or shortening the DC-T-cell interface down to as low as 10 nm, which enhances T cell activation (Du et al., 2023) and further supports the role of intermembrane spacing in the regulation of T cell activation by DCs.

Unlike the well-organized SMAC structure found in the immune synapse formed from the natural interaction between the TCR and pMHC, the synapse created when a CAR interacts with its target is a more disorganized, punctate-like structure. Yet, the same spacing requirement applies to CAR T cell activation. Increasing the length of the hinge in a CAR leads to less exclusion of CD45, reduced phosphorylation of CD3ζ and Erk, and consequently, lower cytokine production and T cell activation (Xiao et al., 2022). As a result, the antitumor efficacy is significantly reduced when the CAR possesses an extended hinge (Xiao et al., 2022).

## 3 Modulating DC-T cell crosstalk for therapeutic development

### 3.1 Customize the DC-T cell crosstalk via vaccination

Vaccines, from conventional attenuated vaccines to subunit vaccines and the most advanced nucleic acid-based vaccines (Figure 1), stand as a cornerstone in public health, aiming at disease prevention and control (Rémy et al., 2015; Pardi et al., 2018; Heidary et al., 2022). Vaccination is a classic and widely used tool to modulate DC crosstalk with other effector immune cells such as T and B cells and prime these against foreign antigens (Clem, 2011). Vaccines are usually formulated with an antigen and an immune-stimulating molecule, termed adjuvant (Facciolà et al., 2022). The traditional adjuvant alum selectively triggers a Th2 response and is inclined to more efficiently elicit a humoral response during vaccination (HogenEsch, 2013). Peptide or nucleic acid vaccines that are well-designed and combined with an effective adjuvant promoting Th1 responses can more effectively induce the preferred type of cellular immunity (Korsholm et al., 2010; Rapaka et al., 2021).

To enable efficient vaccine delivery to the LNs, vaccines are often developed in form of nanoparticles (NPs), LN-targeting polymers, or DC-directed antibody-antigen conjugates (**Figure 1**) (Macri et al., 2016; Irvine et al., 2020). After encountering vaccine antigens and adjuvants, DCs become activated and process the vaccine antigen intracellularly into small peptides of defined lengths which, subsequently, are complexed with MHC class I and MHC class II (Wieczorek et al., 2017). Hereafter, pMHCs are transported to the DC surface to elicit antigen-specific T cell responses via pMHC-TCR interaction, the key axis of DC-T cell crosstalk (Hivroz et al., 2012). Of note, both cDC1 and cDC2 can present exogenous antigens in complex with MHCII to prime CD4 T helper cells (Ferris et al., 2020; Cabeza-Cabrerizo et al., 2021). However, only cDC1 cells have the distinct ability, known as cross-presentation (Joffre et al., 2012), which enables antigen escape from the endosome into the cytoplasm for proteasome processing. This process allows peptide transportation into the endoplasmic reticulum for complexing with MHCI, leading to the priming of antigen-specific cytotoxic CD8 T cells (Embgenbroich and Burgdorf, 2018). This unique cross-presentation property is intrinsic to cDC1 cells, largely owing to their high-level expression of MHCI pathway genes (e.g., TAP1, TAP2, calreticulin) (Dudziak et al., 2007).

In contrast to traditional vaccines, *ex vivo* engineering of DCs optimizes their crosstalk with T cells, offering another promising alternative to prime T cell responses. Immature DCs exhibit a pronounced phagocytic ability, which decreases upon maturation (Kim and Kim, 2019). This characteristic enables *ex vivo* loading of antigens onto patient-derived DCs and reinfusion of these cells as cell-based vaccines (Tacken et al., 2007). The FDA-approved Provenge, composed of GM-CSF and prostatic acid phosphatase (PAP) fusion protein, exemplifies this approach (Cheever and Higano, 2011). For vaccine preparation, monocytes were differentiated into immature DCs (moDCs), matured with pro-inflammatory cytokines, and loaded with antigens through electroporation, viruses, or peptide incubation (Santos and Butterfield, 2018). The effectiveness of DC vaccines varies with maturation and antigen-loading methods (Santos and Butterfield, 2018). Autologous moDCs electroporated with TriMixDC-MEL mRNA (encoding TLR4, CD40L, CD70) show rapid maturation and antitumor activity in a Phase-II study in patients with pretreated advanced melanoma (Wilgenhof et al., 2016). Equadrito et al. developed a chimeric extracellular vesicle (EV)-internalizing receptor (EVIR) technology to enhance DC’s ability to capture cancer-derived extracellular vesicles (Squadrito et al., 2018). EVIR-expressing DCs efficiently deposited OVA^+^MC38 tumor-derived EVs on their surface via cross-dressing and enhanced priming of SIINFEKL-specific OT1 CD8 T cells (Squadrito et al., 2018). In addition, genetically introducing chemokine receptor-7 gene (CCR7) into DCs with a RGD fiber-mutant adenovirus vector (AdRGD) endowed DCs with improved capacity of migrating into regional lymphoid tissues. Upon intradermal injection in mice, these CCR7/DCs accumulate in draining lymph nodes approximately 5.5 times more efficiently than control DCs (Okada et al., 2005). A preclinical study (Yang et al., 2006) and a phase I clinical trial (Lee et al., 2017) of *in situ* administrated DCs, which were genetically modified to overexpress CCL21 (CCL21-DC), demonstrated increased tumor antigen presentation, leading to systemic antitumor immunity.

### 3.2 CAR-T vaccines: an MHC-independent approach crosstalk

Although conventional vaccines and immunotherapies rely on the presentation of antigens by MHC molecules to initiate an immune response, MHC-independent vaccines make it possible to boost engineered T cells, like CAR T cells, in the context of adoptive cell therapy. A CAR T-boosting amphiphile vaccine (amph-vax) was created by conjugating the cognate ligand of the CAR to an albumin-binding lipid polymer (Ma et al., 2019). Following parenteral injection, the amph-vax molecules “hitchhike” serum albumin and are concentrated in the draining LNs where the vaccine takes effect (Liu et al., 2014). Upon arrival, amph-vax molecules directly incorporate into the APC membrane and are subsequently presented to CAR T cells together with co-stimulatory and supporting cytokines, leading to marked enhancement of CAR T expansion, polyfunctionality, memory development, tumor infiltration, and anti-tumor efficacy (Ma et al., 2019). This CAR-dependent vaccination approach represents a novel paradigm for engineering DC-T cell crosstalk, independent of MHCs. In a subsequent study, Ma and colleagues demonstrate the capacity of the vaccine-boosted CAR T cells to crosstalk with the host immune system to effectively reject tumors with antigen heterogeneity (Ma et al., 2023a). Amph-vax boosting of CAR T cells trigger potent priming of endogenous anti-tumor T cells via a process known as antigen spreading which is critically dependent on CAR T cell-derived IFN-γ. Amph-vax augmented CAR T cell therapy effectively shrinks malignant brain tumors that have up to 20% antigen-negative tumor cells, offering a clinically applicable approach for treating solid tumors with pre-existing antigen heterogeneity.

A similar vaccination approach was developed using lipoplexes to deliver modified mRNA molecules (RNA-LPX) encoding the cognate CAR ligand (Reinhard et al., 2020). Using a CAR targeting claudin 6 (CLDN6), a tight junction protein, as a model, Sahin and colleagues demonstrate that intravenous administration of CLDN6 RNA-LPX can efficiently transfect APCs in the spleen and amplify adoptively transferred CLDN6 CAR T cells in both NSG mice and immunocompetent mice (Reinhard et al., 2020), resulting in marked control of CLDN6-expressing tumor progression. Notably, CLDN6 RNA-LPX showed some promises in boosting CLDN6-directed CAR T cells without overt toxicity in a recent phase 1 clinical trial (Mackensen et al., 2023), highlighting the potential of combining vaccines and CAR T cell therapy against solid tumors.

### 3.3 Modulating DC-T cell crosstalk with DC-mimics

Motivated by the natural DC-T cell crosstalk mechanisms, NPs and biomaterials have been engineered to mimic the functions of DCs (DC-mimics) and create synthetic crosstalk with T cells for therapeutic applications. DC-mimic therapy holds huge potential in various medical applications, including vaccination, cancer immunotherapy, and the treatment of autoimmune diseases, by effectively directing the immune system’s response in a controlled manner. We discuss examples from a few major categories of DC-mimics, including membrane-coated NPs, DC-derived nanovesicles and DC-mimicking polymers (Figure 1).

NPs coated with DC-derived membranes (MCNPs) have gained significant attention due to their ability to effectively preserve surface markers and functional proteins from DCs (Liu et al., 2019; Cao et al., 2023; Mackensen et al., 2023). MNCPs manufactured with cell membranes from DCs in BALB/C mice were shown to efficiently trigger activation and proliferation of CD4 T cells from C57BL/6 mice, which was not observed with MCNPs generated using a syngeneic C57BL/6 DC membrane (Li et al., 2023). Coating poly (lactic-co-glycolic acid) (PLGA)-NPs with membranes from BMDCs pretreated with murine ID8 ovarian tumor cell lysate demonstrated enhanced capability of activating T cells both *in vitro* and *in vivo* in mice bearing ID8 ovarian cancer (Cheng et al., 2020). An activated mature dendritic cell membrane (aDCM)-coated nanoplatform with rapamycin (RAPA)-loaded poly (lactic-glycolic acid), named aDCM@PLGA/RAPA, efficiently crossed the blood-brain barrier (BBB) and promoted the activation of tumor-infiltrating T cells and NK cells against glioma *in situ* (Ma et al., 2023b). Recently, MCNPs with modularity were developed using membranes from genetically modified DCs (Krishnan et al., 2023). Membranes engineered to express SpyCatcher quickly create a covalent bond with a ligand that carries a SpyTag. This method allows for the customization of cell membranes with particular ligands, receptors, and signaling molecules, thereby granting NPs improved abilities for targeting, modulating the immune system, and homing to specific tissues (Krishnan et al., 2023). Instead of solely relying on natural DC membrane proteins, an orthogonal bioreactive azide group can be metabolically added onto the membrane glycoporteins for further modification of the DC membrane (Xiao et al., 2021). Imiquimod-loaded and azide-DC membrane coated NPs can be readily modified with anti-CD3ε antibody via click chemistry. These NPs exhibited enhanced distribution in LNs to effective stimulated T cells and LN-resident APCs (Xiao et al., 2021). Similarly, biomimetic magnetosomes with dual functions, created by coating magnetic nanoclusters with azide-engineered leukocyte membranes and then decorating them with T-cell stimuli through click chemistry, not only enhance antigen-specific cytotoxic T-cell (CTL) expansion but also direct CTL infiltration into tumors using magnetic resonance imaging and magnetic control (Zhang et al., 2017). Nanovesicles (NVs) such as exosomes derived from antigen-loaded DCs are also an efficient platform to recreate synthetic DC-T cell crosstalk given their retention of MHC-antigen complexes and co-stimulating molecules that are required for stimulating T cells. Exosomes derived from DCs pulsed with tumor antigens (tDC-Exo) induce the activation of tumor-reactive endogenous T cells, leading to improved therapy against solid tumors (Fan et al., 2022). Another example is the recently developed ASPIRE (antigen self-presentation and immunosuppression reversal) NV platform (Liu et al., 2022). ASPIRE NVs are derived from recombinant adenovirus-infected dendritic cells that simultaneously present antigen-specific pMHCIs, anti-PD1 antibodies, and B7 costimulatory molecules. ASPIRE NVs significantly enhance antigen delivery to lymphoid organs, promoting a broad-spectrum T-cell response capable of eradicating established tumors (Liu et al., 2022).

In addition to NPs or NVs bearing DC membranes, engineered NPs or polymers carrying DC-related ligands represent another approach to bridge synthetic DC-T cell crosstalk. These biomaterials were designed to mimic the interactions between DCs and T cells by incorporating well-defined ligands. A range of bioactive materials is presently under development to enhance the *ex vivo* expansion of T cells for adoptive T cell immunotherapy, commonly referred to as artificial antigen-presenting cells (aAPCs) (Eggermont et al., 2014; Wauters et al., 2022; Kang et al., 2023). These aAPCs have been instrumental in improving the *ex vivo* manufacturing of engineered T cells. Examples include conventional dynabeads decorated with anti-CD3 and anti-CD28/41BB antibodies (Eggermont et al., 2014), biocompatible dendritic poly (N-isopropylacrylamide) (PNIPAM) microspheres with surface morphology that mimics natural DCs (Yang et al., 2023), or a recently engineered nanosized immunofilament presenting anti-CD3/pMHC, CD28, and IL-2 (Weiss et al., 2023). Furthermore, NPs can be engineered to mimic the immunoregulatory function of DCs for the treatment of autoimmune diseases. Santamaria and colleagues modified iron oxide NPs with type 1 diabetes (T1D)-specific pMHCs (Tsai et al., 2010). They found that administering these pMHC-NPs into wild-type mice and humanized non-obese diabetic mice can convert naive low-avidity autoreactive CD8^+^ T cells into memory-like autoregulatory T cells that effectively prevent the onset of T1D and restore normoglycemia in diabetic animals (Tsai et al., 2010). Additionally, they note that the induction of autoregulatory T cells is strongly dependent on the density of pMHCs (Singha et al., 2017).

## 4 Conclusion and future perspectives

The Crosstalk between DCs and T cells are central to the establishment of protective immunity or maintaining immune homeostasis. Ample research has revealed the critical signaling molecules, downstream pathways and unique biophysical features involved in the DC-T cell crosstalk. In this review article, we not only covered the natural crosstalk mechanisms between DCs and T cells but also discussed classical and advanced engineering approaches that leveraged these crosstalk mechanisms for tailoring T cell responses for therapeutic interventions.

The comprehensive understanding of DC-T cell crosstalk from decades’ work offers a perfect test ground for modulating T cell responses by indirectly targeting DCs. A myriad of technologies including biomaterials, NPs and genetically engineered immune cells, have been developed to modulate DC-T cell crosstalk to achieve temporospatial regulation. These were exemplified by novel vaccines with preferential lymphoid organ homing ability and various DC-mimics simulating the characteristics of defined DC subsets. In addition to targeting DCs in the lymphoid organs, the recent discovery of complex crosstalk between intratumoral DCs and both CD4 and CD8 T cells during effective immunotherapy highlighted the need for targeted modulation of the DC-T cell crosstalk within the tumor microenvironment in order to achieve superior therapeutic responses. Furthermore, biomaterials such as synthetic vaccines designed to bridge genetically engineered CAR T cells with lymph node-resident DCs offer a possibility to integrate powerful genetic engineering approaches which further tailor DC-T cell crosstalk for desired therapeutic applications. Finally, given that immune cells interact within a highly intricate network, strategies that target the crosstalk between DCs and T cells in the lymph node are likely to affect the wider immune system via signals from cytokines and chemokines. The development of effective immunotherapies is leveraging both technological advancements and a deeper comprehension of the crosstalk mechanisms between DCs and T cells. These investigations have the potential to pioneer novel avenues in immunotherapy research.
